# Leukoplakia and erythroplakia in youngers versus older individuals: a clinicopathological retrospective study

**DOI:** 10.4317/medoral.26659

**Published:** 2024-08-01

**Authors:** Uarlei N Porto, Natalia K Laureano, Natália S dos Santos, Amanda Z Rodrigues, Camila A Ferri, Taiane B de Lima, Pantelis V Rados, Laura C Hildebrand, Marco Antônio T Martins, Vinicius C Carrard, Fernanda Visioli

**Affiliations:** 1 Department of Oral Pathology, School of Dentistry, Universidade Federal do Rio Grande do Sul, Porto Alegre, Rio Grande do Sul, Brazil; 2 Oral Medicine Unit, Otorhinolaryngology Service, Hospital de Clínicas de Porto Alegre, Porto Alegre, Rio Grande do Sul, Brazil; 3 Experimental Research Center, Hospital de Clínicas de Porto Alegre, Porto Alegre, Rio Grande do Sul, Brazil

## Abstract

**Background:**

The incidence of oral cancer has exhibited a rise within the young population. Considering that oral potentially malignant disorders (OPMDs) can precede the development of oral cancer, it is imperative to conduct studies in this particular younger population. This study aimed to evaluate the frequency and conduct a comparative analysis of the clinical-demographic characteristics of OPMDs in two distinct age groups.

**Material and Methods:**

A retrospective analysis was conducted with patients diagnosed with leukoplakia, erythroplakia, and leukoerythroplakia between 1965 and 2020. The individuals were categorized into two groups: those aged up to 40 years (Group Younger) and those aged 41 years and above (Group Older).

**Results:**

A total of 640 lesions were subjected to analysis. Among these, patients aged up to 40 years constituted 10.63% of the sample, however, this proportion decreased significantly to 6.9% between 2010 and 2020. A predominant male representation was observed in both groups, with white lesions being the most common in both as well. However, the frequency of red or mixed lesions was significantly higher (*p*=0.034) in the older group, along with a higher prevalence of dysplastic lesions (26.9% versus 11.8%, *p*=0.01). Moreover, the older group exhibited a relatively higher percentage of smokers/ex-smokers (78.6%), compared to the younger group (61.5%, *p*=0.085) and alcohol consumers/ex-consumers (54.9% versus 22.7%, *p*=0.028). Elderly individuals exhibited an unfavorable progression (*p*=0.028). However, a logistic regression analysis identified as significant variables associated with malignant transformation, the presence of epithelial dysplasia, and red lesions diagnosed as erythroplakia.

**Conclusions:**

A declining frequency of OPMDs in young adults was observed over the years, whereas in older adults, these disorders exhibited an unfavorable progression.

** Key words:**Oral cancer, age, oral potentially malignant disorders, malignant transformation.

## Introduction

Oral squamous cell carcinoma (OSCC) stands as the most prevalent malignant tumor in the oral cavity and its main etiological factors are linked to tobacco and alcohol consumption (1). Historically, OSCC has demonstrated a higher prevalence in white male individuals aged over 50 years (2). Nevertheless, recent research has provided evidence that the incidence of OSCC in younger patients has experienced a noticeable increase in recent decades (3).

The rise in OSCC incidence among young individuals has affected both men and women. However, there are conflicting results regarding the frequency of exposure to tobacco and alcohol in this younger population (4,5). It has been suggested that other factors, such as genetic predisposition, systemic conditions, dietary habits, environmental exposures, and infection by the human papillomavirus (HPV), could directly contribute and play a crucial role in the development of OSCC in young adults (4-6).

Oral potentially malignant disorders (OPMDs) have been recognized to potentially precede the development of OSCC. Among the epithelial lesions with a potentially malignant nature, leukoplakia stands as the most prevalent, characterized by a predominantly white appearance (7). Leukoplakia has prevalence of approximately 2% within the general population, and a malignant transformation rate of 1.1% to 40.8% of cases (8). Erythroplakia is another OPMDs defined as “predominantly fiery red patch that cannot be characterized clinically or pathologically as any other definable disease” (7). Erythroplakia is often considered to carry an unfavorable prognosis due to its high frequency of malignant transformation, close to 30-50%. Lesions with mixed white and red compartments are named as leukoerythroplakia (9).

Given the evolving epidemiological trends in oral cancer, often preceded by OPMDs, there's a pressing need for further research to determine if OPMDs have also shifted in their epidemiological patterns in recent years. Hence, this study aims to evaluate the frequency of younger patients diagnosed with oral leukoplakia and erythroplakia spanning over five decades. Additionally, the study seeks to characterize these lesions in younger individuals and compare them with older age groups.

## Material and Methods

- Sample and data collection

A retrospective analysis was conducted with histological slides of individuals diagnosed with leukoplakia, erythroplakia, and leukoerythroplakia over the past 5 decades (January 1965 to December 2020). Data were gathered from registers, medical and biopsy records from the Oral Medicine service of the Hospital de Clínicas de Porto Alegre (Brazil), and from the Oral Pathology service at Universidade Federal do Rio Grande do Sul (Brazil).

Cases with an absence of more than 50% of the necessary information regarding the studied variables, as well as lesions with incompatible histopathological diagnosis, were excluded from the analysis. All samples were evaluated according to World Health Organization criteria. Microscopic diagnosis of squamous cell carcinoma (SCC) was included solely in cases where the clinical presentation of the lesions aligned with OPMD rather than ulcerated lesions.

Individuals up to 40 years of age (6,10,11) were classified as young at the time of diagnosis (Group Younger), while adults aged 41 and above were categorized as the older age group (Group Older). The variables analyzed were age, gender, skin color, smoking and alcohol consumption, lesion location, clinical appearance of the lesion, clinical diagnosis, microscopic diagnosis, type of biopsy performed, date of biopsy, outcome (favorable or unfavorable evolution) and occurrence of malignant transformation. Regarding anatomical sites, lesions located at the floor of the mouth, the edge of the tongue, and the soft palate were considered high-risk locations, while all other regions were categorized as low-risk locations (12,13). The progression of the lesions was classified as "favorable" when there was no alteration or in cases of clinical improvement after an incisional biopsy, or when there was no recurrence after an excisional biopsy. The progression of the lesions was classified as "unfavorable" when there was clinical deterioration, recurrence, development of new lesions, or malignant transformation. It was only considered malignant transformation if it occurred at least 6 months after previous biopsy in order to avoid misdiagnosis.

- Data analysis and statistics

Statistical analysis was carried out using SPSS software (Statistical Package for the Social Sciences version 23.0, IBM). Quantitative variables were described based on absolute and relative frequencies. Descriptive and bivariate analyses were conducted to assess the association between the independent variables and the outcome, utilizing Fisher's Exact Test or Chi-Square Tests (*p*<0.05). Variables associated with the lesion evolution outcome were identified using binary logistic regression.

## Results

- OPMDs characteristics in younger versus older patients

This study identified 785 reports of clinical lesions. Sixty-two cases were excluded due to missing data, and 83 cases were excluded because they were not confirmed microscopically. Finally, 640 lesions in 522 patients (Table 1) were included, with 572 (89.37%) lesions in the older group and 68 (10.63%) lesions in the younger group. In both groups, the majority of patients were male. Notably, the frequency of lesions among young patients showed a significant decrease over time (*p*<0.0001), declining from 15.28% in the decade from 2001 to 2010 to 6.92% in the following decade (2011 to 2020).

In many cases, information regarding habits was missing (Table 1); however, based on the available data, the majority of individuals reported being smokers in both groups. Notably, the number of never smokers in the Younger Group was higher compared to the Older Group (38.5% and 21.4%, respectively), although not statistically significant (*p*=0.085). Concerning alcohol consumption, among the reported cases, it was observed a higher frequency of drinkers in the Older Group (*p*=0.028) compared to the Younger (36.8% versus 18.2%, respectively). Considering the type of biopsy performed, excisional biopsies were more commonly performed in the Younger Group (52.1%), while in the Older Group, most patients underwent incisional biopsies (65.6%) (*p*=0.024)

**Figure 1:**
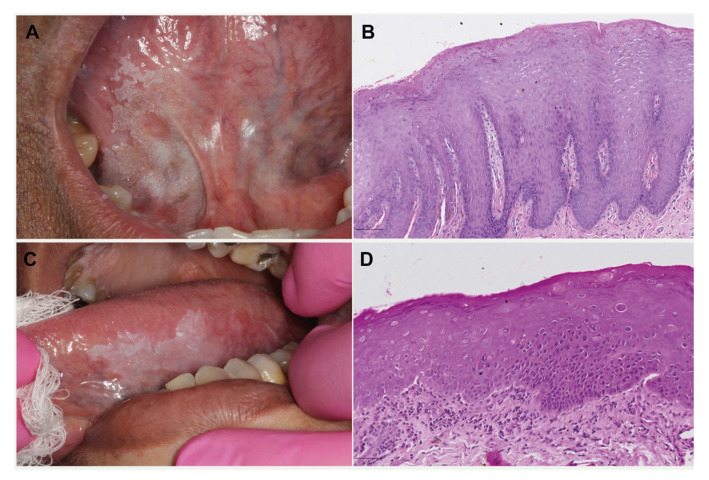
Representative cases of each group. A) Younger group: Clinical image of leukoplakia on the ventral tongue in a 36-year-old non-smoker male. B) Microscopic image of the lesion shown in A, revealing epithelial hyperplasia and hyperkeratosis. C) Older group: Clinical image of leukoerythroplakia on the border of the tongue in a 64-year-old smoker female. D) Incisional biopsy of the lesion shown in C revealed epithelial dysplasia.

In both groups, white lesions were predominantly observed, but the frequency of white lesions was significantly higher among the Younger Group compared to the Older Group, which presented an increase in red and mixed lesions (*p*=0.034). Accordingly, the clinical diagnosis of leukoplakia was estimated in 97.1% and 87.6% of cases comparing youngs and olders, respectively. Most of the lesions observed in both groups were located in low-risk sites (65.2% and 57.2%). Images of representative cases of each group are available in Fig. 1.

Among the Younger Group, only 11.8% of the patients were diagnosed with epithelial dysplasia, whereas in the Older Group this proportion increased to 22.02% of the cases (*p*=0.01). Specifically, in the Older Group, the presence of SCC was detected in 4.8% (*n*=28) of the sample, with no cases of malignancy being evidenced in the Younger Group (Table 2).

- OPMDs evolution and risk for malignant transformation in younger and older patients

Out of the initially identified 522 patients, follow-up information was available for 234. Most of the lesions in both groups remained sTable over time (63.6% and 41.7% in the Younger and Older Groups, respectively).

Older patients exhibited a higher frequency of unfavorable evolution (39.5%) and 15.7% underwent malignant transformation. In contrast, for the Younger Group only 18.2% of lesions presented an unfavorable evolution and only one case underwent malignant transformation. The Older Group displayed a statistically significantly worse evolution (*p*=0.028) when compared to the Younger Group (Table 2).

The studied variables were included in a logistic regression model to determine the relative risk concerning the unfavorable evolution of the lesions. The analysis revealed that a dysplastic histopathological diagnosis acted as a risk factor for unfavorable evolution (OR=2.987, 95% CI 1.612-5.533, *p*=0.001). Similarly, being female was also found to be a risk factor (OR=1.755, 95% CI 1.039-2.963, *p*=0.048), as well as being over 41 years old (OR=3.280, 95% CI 1.039 - 10.354, *p*=0.043). On the other hand, being a smoker is a protective favor (OR=0.376, 95% CI 0.173-0.816, *p*=0.013) (Table 3).

The risk analysis for malignant transformation revealed that the histopathological diagnosis of epithelial dysplasia increased the likelihood of malignant transformation by 9.8 times (OR=9.861, 95% CI 4.157 - 23.394, *p*=0.001). Having a red lesion increased the risk by 5.1 times (OR=5.152, 95% CI 1.639 - 16.196, *p*=0.005), and being diagnosed with erythroplakia increased the risk by 4.6 times (OR=4.673, 95% CI 1.398 - 15.616, *p*=0.012). Furthermore, being a smoker or former smoker decreased the risk of malignant transformation (OR=0.7 and OR=0.16, respectively) (Table 3).

## Discussion

Despite the rising incidence of OSCC in young individuals and the extensive characterization studies of OMPDs conducted over the years, it remains uncertain whether a similar trend is also occurring with the incidence of OMPDs (3,14). Contrary to what has been observed in OSCC (3), our findings revealed a decline of OPMDs frequency in youngers overtime. This profile aligns with findings from other studies, which also indicate a low frequency of oral leukoplakias in young patients (11). The decrease in OPMDs frequency in this particular population can be attributed to several factors. One reason may be the lack of early diagnosis or misdiagnosis of OPMDs, where they might be mistaken for reactional keratotic lesions or other similar conditions. The underdiagnosis of OPMDs in young patients might result from the perception that this age group is not considered at high risk for such disorders. Furthermore, routine examination of the oral mucosa is not consistently carried out by most general practitioner dentists, which could also contribute to the reduced detection of OPMDs in young individuals.

Smoking and alcohol habits are well-established risk factors strongly associated with the development of OMPD and OSCC (5,7). Our study observed a majority of smoking history in both groups. Although there were more never-smokers in the young group, no statistically significant difference was detected. Considering alcohol status, younger patients showed a significant amount of never drinking history. Similar findings were reported in the meta-analyses conducted by Roza *et al*. (2021) and Aguirre-Urizar *et al*. (2021), suggesting that other risk factors, despite alcohol, play a significant role in young patients prone to oral carcinogenesis (8,11).

A slightly higher frequency was observed for OPMDs affecting men in both age groups. This similarity in the distribution of sex observed in the development of OPMDs can be attributed to the changing social behavior and increased engagement of women in carcinogenic habits more frequently (15). However, a different pattern of prevalence was noted in the systematic review of OPMDs in young people conducted by Roza *et al*. (2020), which identified a pronounced male predominance in 87.8% of patients (11). This distinct proportion compared to our findings in young patients might be due to the systematic review encompassing countries and continents with varying habits and cultures (China, Iran, Taiwan, and South Africa), while our study assessed a more homogenous sample from the southern region of Brazil.

Regarding OPMDs clinical diagnosis, leukoplakia emerged as the most prevalent lesion, consistent with findings from other studies on the same subject (14). In this study, white lesions displayed a statistically significant higher frequency in the group of adults under 40 years old, whereas red or mixed lesions were more commonly detected in older adults. In agreement, it was observed a higher level of epithelial dysplasia in the group with higher age (22.02%) when compared to the younger (11,8%). This data may also be influenced by lesion locations, since a higher number of lesions in low-risk locations (65.2% in youngers versus 57.2% in elders), which may reflect in the lower frequency of epithelial dysplasia in the histopathological results of both groups (11.8% in Younger Group and 26.9% in the Older Group). Similar findings were observed by Jaber *et al*. (2003) and Speight *et al*. (2018), where lesions found in high-risk locations, such as the tongue, floor, and ventral portion, showed a higher frequency of epithelial dysplasia in their respective samples (16,17).

The OPMDs here evaluated were located in different sites of the oral mucosa, and due to their potential for malignancy, biopsy is mandatory (18). In this study, there was a predominance of excisional biopsies in younger patients (52.1%), whereas in the older group, a significantly higher number of participants underwent incisional biopsies (65.6%). The greater number of excisional biopsies in the Younger Group can be explained by professionals considering the age of these patients when deciding between minor or major procedures like excisional biopsy. Additionally, factors such as the lower frequency of comorbidities, location, size of the lesion, clinical appearance, and diagnosis, may also justify the decision for complete removal in the younger age group.

Moreover, the lesions observed in young patients exhibited better evolution compared to those found in the group aged over 40 years, which displayed a worse clinical evolution. Age is considered an evident factor influencing the worse evolution of oral OPMDs, owing to the accumulation of various molecular and oncogenic events over time. These events include alterations in the immune system, persistent exposure to carcinogenic risk factors leading to cellular damage and genetic mutations that promote the progression of these oral lesions (17,19).

In our sample, the risk factors considered significant for unfavorable evolution were being female, over 41 years of age, and having a histopathological diagnosis of epithelial dysplasia. However, focusing specifically on malignant transformation, the only factors associated with an increased risk of malignant transformation were presence of red lesions, presence of epithelial dysplasia, and being non-smokers. Similar findings can be observed in the meta-analysis conducted by Aguirre-Urizar *et al*. (2021), which revealed that the risk of malignant transformation in cases of lesions with epithelial dysplasia is 23.8 times higher (8). The same can be observed regarding the age factor, as their meta-analysis showed that most cases of malignant transformation occurred in people over 50 years old (82.5%), which is consistent with the data observed in our sample, where majority of cases with malignant transformation occurred in older individuals (97%).

Although there is no clear evidence or explanation for the predominance of malignant transformation of OPMDs in women, the meta-analysis by Warnakulasuriya *et al*. (2016) demonstrated that women have a 1.65 times greater risk of malignant transformation compared to men. Huber *et al*. (2002) and Suba *et al*. (2007) suggest that this result may be attributed to genetic, epigenetic, and hormonal dysregulations, which could be associated with the process of oral carcinogenesis in women with OMPDs (20,21). These findings were also confirmed by other studies (17,22).

We acknowledge certain limitations in our study, which are inherent to retrospective research. The clinical and histopathological diagnosis was conducted by various professionals over the decades, therefore lacking standardized criteria, and incomplete sociodemographic information or medical records over the years. Additionally, this is not a multicenter study, and therefore, generalization of findings to other populations should be performed cautiously. Nonetheless, a significant strength of our study is the large and representative sample observed over a considerable period of 55 years (1965 to 2020), enabling comprehensive evaluation of these patients and establishment of meaningful outcomes.

From the analysis of this sample, we can deduce that the frequency of young patients diagnosed with OPMD is lower compared to the group of older adults in recent years. Additionally, it was observed that in adults over 40 years old, the OMPDs may exhibit a worse clinical evolution.

## Figures and Tables

**Table 1:** Sociodemographic characteristics of the sample.

Characteristics		Group I Up to 40 years-old		Group II Above 41 years-old		*p*
		N	%	N	%	
Gender	Female	30	44.1	261	45.6	0.827
	Male	38	55.9	311	54.4	
Age range (mean ± SD)		18 - 40 (33.7± 5,79)		41 - 87 (58.89±11,21)		
Race	White	52	82.5	481	88.1	0.462
	Non-white	8	12.7	42	7.7	
	Others	3	4.8	23	4.2	
	Not informed	5	-	31	-	
Year of diagnosis	1965-2000	14	28	36	72	< 0.0001
	2001-2010	24	15.28	133	84.72	
	2011-2020	30	6.92	403	93.08	
Smoke status	Smoker	16	41.0	219	55.2	0.085
	Former smoker	8	20.5	93	23.4	
	Never smoker	15	38.5	85	21.4	
	Not informed	29	-	175	-	
Alcohol status	Drinker	4	18.2	120	36.8	0.028
	Former drinker	1	4.5	59	18.1	
	Never drinker	17	77.3	147	45.1	
	Not informed	46	-	246	-	
Biopsy	Incisional	23	47.9	337	65.6	0.024
	Excisional	25	52.1	177	34.4	
	Not informed	20	-	58	-	

*p*, chi-square.

**Table 2:** Clinicopathological features, anatomical site, and evolution of the lesions according to the group.

Clinicopathological features, anatomical site, and evolution of the lesions		Group I Up to 40 years-old		Group II Above 41 years-old		*p*
		N	%	N	%	
Clinical appearance	White	65	95.6	485	85.7	0.034
	Red / White-red	3	4.4	81	14.3	
	Not informed	0	-	6	-	
Clinical diagnosis	Leukoplakia	66	97.1	501	87.6	0.121
	Erythroplakia	1	1.5	17	3.0	
	Leukoerythroplakia	1	1.5	54	9.4	
Histology	Non-dysplastic	60	88.2	418	73.1	0.010
	Epithelial dysplasia	8	11.8	126	22.02	
	SCC	0	0	28	4.8	
Grouped anatomical site	Low-risk	43	65.2	318	57.2	0.269
	High-risk	23	34.8	238	42.8	
	Not informed	2	-	16	-	
Evolution	No recurrence	1	9.1	22	9.9	-
	Clinical improvement	1	9.1	7	3.1	
	Clinical worsening	0	0	33	14.8	
	Relapse	0	0	10	4.5	
	New lesions	2	18.2	45	20.2	
	Malignant transformation	1	9.1	35	15.7	
	Unchanged	6	54.5	71	31.8	
	Total	11	100	223	100	
Grouped evolution	Favorable	8	72.7	100	44.8	0.028
	Unfavorable	2	18.2	88	39.5	
	Malignant transformation	1	9.1	35	15.7	

*p*, chi-square.

**Table 3:** Logistic Regression to identify factors associated with unfavorable evolution and malignant transformation of lesions.

Outcome		Unfavorable evolution			Malignant transformation		
		OR	[IC95%]	*p*	OR	[IC95%]	*p*
Histology	Non-dysplastic	ref	-	-	ref	-	-
	Dysplastic	2.987	[1.612-5.533]	0.001	9.861	[4.157 - 23.394]	0.0001
Age	Up to 40 years	ref	-	-	ref	-	-
	Over 41 years	3.280	[1.039-10.354]	0.043	1.862	[0.231 - 15.007]	0.559
Sex	Male	ref	-	-	ref	-	-
	Female	1.755	[1.039-2.963]	0.048	1.696	[0.830 - 3.469]	0.148
Ethnicity	Others	ref	-	-	ref	-	-
	White	1.536	[0.401-5.880]	0.531	0.339	[0.030 - 3.850]	0.383
	Black	0.750	[0.107-5.238]	0.772	0.286	[0.012 - 6.914]	0.441
Smoking	No	ref	-	-	ref	-	-
	Former smoker	0.487	[0.216-1.098]	0.083	0.160	[0.63 - 0.406]	0.001
	Yes	0.376	[0.173-0.816]	0.013	0.70	[0.25-0.196]	0.001
Alcohol consumption	No	ref	-	-	ref	-	-
	Yes	1.152	[0.551-2.408]	0.885	0.857	[0.271 - 2.714]	0.793
	Former drinker	0.933	[0.380-2.292]	0.631	1.719	[0.553 - 5.346]	0.350
Clinical diagnosis	Leukoplakia	ref	-	-	ref	-	-
	Erythroplakia	1.462	[0.461-4.643]	0.519	4.673	[1.398 - 15.616]	0.012
	Leukoerythroplakia	1.269	[0.647-2.489]	0.488	2.266	[0.977 - 5.255]	0.057
Clinical aspect	White lesion	ref	-	-	ref	-	-
	Red lesion/	1.594	[0.514-4.941]	0.419	5.152	[1.639 - 16.196]	0.005
	Mixed white/Red	1.033	[0.516-2.068]	0.927	1.503	[0.594 - 3.799]	0.389
Biopsy type	Incisional	ref	-	-	ref	-	-
	Excisional	1.466	[0.767-2.804]	0.247	0.700	[0.205 - 2.393]	0.570
Anatomical location	Low risk	ref	-	-	ref	-	-
	High risk	1.204	[0.713-2.033]	0.487	1.231	[0.591 - 2.563]	0.578

OR = Odds ratio; Ref = reference categories; Oucome: bad evolution.

## References

[1] Gormley M, Creaney G, Schache A, Ingarfield K, Conway DI (2022). Reviewing the epidemiology of head and neck cancer: definitions, trends and risk factors. Br Dent J.

[2] Daroit NB, Martins LN, Garcia AB, Haas AN, Maito FLDM, Rados PV (2023). Oral cancer over six decades: a multivariable analysis of a clinicopathologic retrospective study. Braz Dent J.

[3] Hussein AA, Helder MN, de Visscher JG, Leemans CR, Braakhuis BJ, Forouzanfar T (2017). Global incidence of oral and oropharynx cancer in patients younger than 45 years versus older patients: A systematic review. Eur J Cancer.

[4] van Monsjou HS, Wreesmann VB, van den Brekel MWM, Balm AJM (2013). Head and neck squamous cell carcinoma in young patients. Oral Oncol.

[5] Toporcov TN, Znaor A, Zhang ZF, Yu GP, Winn DM, Wei Q (2015). Risk factors for head and neck cancer in young adults: a pooled analysis in the INHANCE consortium. Int J Epidemiol.

[6] Llewellyn CD, Johnson NW, Warnakulasuriya KA (2001). Risk factors for squamous cell carcinoma of the oral cavity in young people--a comprehensive literature review. Oral Oncol.

[7] Warnakulasuriya S, Kujan O, Aguirre-Urizar JM, Bagan JV, González-Moles MÁ, Kerr AR (2021). Oral potentially malignant disorders: A consensus report from an international seminar on nomenclature and classification, convened by the WHO Collaborating Centre for Oral Cancer. Oral Dis.

[8] Aguirre-Urizar JM, Lafuente-Ibáñez de Mendoza I, Warnakulasuriya S (2021). Malignant transformation of oral leukoplakia: Systematic review and meta-analysis of the last 5 years. Oral Dis.

[9] Iocca O, Sollecito TP, Alawi F, Weinstein GS, Newman JG, De Virgilio A (2020). Potentially malignant disorders of the oral cavity and oral dysplasia: A systematic review and meta-analysis of malignant transformation rate by subtype. Head Neck.

[10] Mneimneh WS, Xu B, Ghossein C, Alzumaili B, Sethi S, Ganly I (2021). Clinicopathologic Characteristics of Young Patients with Oral Squamous Cell Carcinoma. Head Neck Pathol.

[11] Roza ALOC, Kowalski LP, William WN Jr, de Castro G Jr, Chaves ALF, Araújo ALD (2021). Latin American Cooperative Oncology Group-Brazilian Group of Head and Neck Cancer. Oral leukoplakia and erythroplakia in young patients: a systematic review. Oral Surg Oral Med Oral Pathol Oral Radiol.

[12] Napier SS, Speight PM (2008). Natural history of potentially malignant oral lesions and conditions: an overview of the literature. J Oral Pathol Med.

[13] Zhang L, Cheung KJ Jr, Lam WL, Cheng X, Poh C, Priddy R (2001). Increased genetic damage in oral leukoplakia from high risk sites: potential impact on staging and clinical management. Cancer.

[14] Mello FW, Miguel AFP, Dutra KL, Porporatti AL, Warnakulasuriya S, Guerra ENS (2018). Prevalence of oral potentially malignant disorders: A systematic review and meta-analysis. J Oral Pathol Med.

[15] Sturgis EM, Cinciripini PM (2007). Trends in head and neck cancer incidence in relation to smoking prevalence: an emerging epidemic of human papillomavirus-associated cancers?. Cancer.

[16] Jaber MA, Porter SR, Speight P, Eveson JW, Scully C (2003). Oral epithelial dysplasia: clinical characteristics of western European residents. Oral Oncol.

[17] Speight PM, Khurram SA, Kujan O (2018). Oral potentially malignant disorders: risk of progression to malignancy. Oral Surg Oral Med Oral Pathol Oral Radiol.

[18] Warnakulasuriya S (2009). Global epidemiology of oral and oropharyngeal cancer. Oral Oncol.

[19] Ilhan B, Epstein JB, Guneri P (2019). Potentially premalignant disorder/lesion versus potentially premalignant patient: Relevance in clinical care. Oral Oncol.

[20] Huber JC, Schneeberger C, Tempfer CB (2002). Genetic modelling of the estrogen metabolism as a risk factor of hormone-dependent disorders. Maturitas.

[21] Suba Z (2007). Gender-related hormonal risk factors for oral cancer. Pathol Oncol Res.

[22] Warnakulasuriya S, Ariyawardana A (2016). Malignant transformation of oral leukoplakia: a systematic review of observational studies. J Oral Pathol Med.

